# Exogenous lipoid pneumonia: an important cause of interstitial lung disease in infants

**DOI:** 10.1002/rcr2.356

**Published:** 2018-08-07

**Authors:** Diana Marangu, Komala Pillay, Ebrahim Banderker, Diane Gray, Aneesa Vanker, Marco Zampoli

**Affiliations:** ^1^ Department of Paediatrics and Child Health University of Nairobi Nairobi Kenya; ^2^ Division of Paediatric Pulmonology, Department of Paediatrics and Child Health and Medical Research Council Unit on Child and Adolescent Health University of Cape Town Cape Town South Africa; ^3^ Division of Pathology, Department of Paediatrics and Child Health University of Cape Town Cape Town South Africa; ^4^ Division of Radiology, Department of Paediatrics and Child Health University of Cape Town Cape Town South Africa

**Keywords:** Africa, Children’s interstitial lung disease (chILD), *Mycobacterium abscessus*, oil

## Abstract

Exogenous lipoid pneumonia (ELP), an important cause of interstitial lung disease, often goes unrecognized. We conducted a retrospective study of children with histologically confirmed ELP at Red Cross Children’s Hospital, South Africa. Twelve children of Zimbabwean heritage aged 2.1–10.8 months were identified between 2012 and 2017. Repeated oral administration of plant‐based oil for cultural reasons was reported by 10 of 11 caregivers. Cough (12/12), tachypnoea (11/12), hypoxia (9/12), and diffuse alveolar infiltrates on chest radiography (12/12) were common at presentation. Chest computed tomography revealed ground‐glass opacification with lower zone predominance (9/9) and interlobular septal thickening (8/9). Bronchoalveolar lavage specimens appeared cloudy/milky, with abundant lipid‐laden macrophages and extracellular lipid on Oil‐Red‐O staining (12/12), with polymicrobial (6/12) and Mycobacterium abscessus (2/12) co‐infection. Antibiotics, systemic corticosteroids, and therapeutic lavage were interventions in all eight and five patients, respectively. Clinicians should consider ELP in children with non‐resolving pneumonia in settings with similar practices.

## Introduction

Exogenous lipoid pneumonia (ELP) is considered a rare disorder caused by inhalation or aspiration of mineral, plant‐based, or animal oils 1, 2. It results from a foreign body type of inflammatory reaction due to the presence of lipid material in the lung parenchyma initiating cellular and humoral defence mechanisms 3, 4. Populations at risk include the elderly, children, and those with an underlying swallowing dysfunction or neurological disorder resulting in an unprotected airway. In addition, forced ingestion in the recumbent position in infants and young children who actively refuse oil may result in gagging and aspiration of the oil 5. Common ailments for which oil has been used medically or culturally include constipation, colic, and nasal stuffiness 6.

Oil administration may be a common practice that is not recognized to be potentially dangerous by caregivers or medical professionals. This history may not be forthcoming from caregivers unless health workers probe for it explicitly, leading to misdiagnosis, treatment delay, and a missed opportunity to prevent ongoing oil aspiration 2. The aim of our study was to describe the clinical–radiological–pathological pattern of ELP in children in the South African context.

## Case Series

Following ethical approval from the University of Cape Town Human Research Ethics Committee*,* we conducted a retrospective case series at Red Cross Children’s Hospital, a tertiary referral hospital in South Africa.

### Participant Selection

We consecutively selected children aged <18 years with histologically confirmed ELP defined under the following conditions: (1) clinical presentation of persistent or recurrent unexplained pneumonia associated with tachypnoea or hypoxia; (2) radiological evidence of persistent diffuse alveolar infiltrates on chest radiography or chest computed tomography (CT); (3) extracellular lipid and lipid‐laden macrophages in bronchoalveolar lavage (BAL) and/or frozen section lung biopsy; and (4) history of oil administration related to time of presentation/diagnosis.

### Study Procedures

Permission to search patient records including medical folders and contact information was obtained from the hospital management. The lead researcher reviewed medical records of eligible patients and extracted relevant clinical data. Data were captured electronically, anonymized, and recorded in a standard case report form. The study radiologist and pathologist independently reviewed and entered data related to chest radiography, CT findings, BAL, and lung biopsy findings.

Children with suspected interstitial lung disease (ILD) routinely underwent high‐resolution CT utilizing a paediatric‐friendly radiation dosing protocol with controlled ventilation if under the age of six years. Flexible bronchoscopy (Olympus® 2.8 mm) and radiology‐directed BAL was performed in all children under general anaesthesia through a laryngeal mask. Histocytological assessment of BAL and lung biopsy specimens routinely include Oil Red O stain for lipids, periodic acid Schiff stain for glycoprotein exudates, Perls’ Prussian Blue stain for iron, Grocott’s methanamine silver stain for fungi, and Ziehl‐Neelsen stain for acid fast bacilli. Processing and examination of BAL and lung tissue were conducted according to the European Management Platform for Childhood Interstitial Lung Disease protocols 7. Partial therapeutic lung lavage (using 2.8 mm Olympus® flexible bronchoscope) with 200–300 mL 0.9% warmed saline, targeting the worst affected regions of the lung, was performed in selected cases where clinically significant hypoxemia and/or symptoms did not resolve spontaneously with medical management and cessation of oil administration in hospital. Repeated therapeutic lavages were performed where needed.

### Clinical Characteristics

Between October 2012 and December 2017, we identified 12 children with ELP as per our study case definition. All children were of Zimbabwean heritage, presenting in infancy (range 2.1–10.8). Dry cough was the main presenting symptom in all children with a duration varying from one day to three months. Common symptoms at presentation of these infants included: tachypnoea (11/12), hypoxia (9/12), fever (4/12), hyperinflation (2/12), and digital clubbing (2/12). Six of 12 children were hospitalized for pneumonia on at least one other occasion besides the episode in which the diagnostic BAL that was conducted provided histocytological evidence of ELP. Underlying risk factors that were documented included gastro‐oesophageal reflux (GOR) confirmed on scintigraphy (3/6), in combination with silent aspiration confirmed on contrast swallow (2/4). Ten of the 11 mothers interviewed confirmed the administration of oil to their children. In five patients, this history was obtained after reviewing BAL findings. Oil administration emerged to be a nearly universal cultural practice by Zimbabweans, even in the Cape Town diaspora. (Table 1).

**Table 1:** Demographic, clinical, and oil exposure characteristics.

Patient ID	Gender	Age diagnosis (months)	Type of oil	Duration; onset; amount and frequency oil administered	Underlying comorbidity and risks for aspiration	Clinical presentation	Duration of symptoms (weeks)	Co‐infections on NPA and/or BAL*
01†	Male	6.1	Sunflower oil	12 months; from day 2 of life; 2.5 mL orally twice daily	GOR‡, no aspiration§	Cough, tachypnoea, hypoxia	0.9	Parainfluenza
02¶	Female	9.8	Not available	Not available	None**	Cough, fever, tachypnoea, hypoxia	12.0	None
03† ^,^ ††	Male	7.5	Olive oil	14 days; from the age of six months; 2.5 mL orally once daily	None**	Tachypnoea, hypoxia	4.0	Enterovirus*, human rhinovirus
04‡‡ ^,^ §§	Male	1.4	Mother denied giving oil	Not known	Resolved renal disease¶¶	Cough, tachypnoea, hypoxia, three prior LRTI hospitalizations	0.3	*Klebsiella pneumoniae* *, human rhinovirus
				Child under the care of grandmother	None**			
				Unconfirmed suspicion that grandmother gave oil				
05***	Male	6.2	Plant‐based “cooking” oil†††	Five months; from day 1 of life; 5 mL orally twice daily	Refused to drink oil‡‡‡	Cough, wheeze, crepitations, two prior LRTI hospitalizations	0.4	Adenovirus*, RSV‐B*, Enterovirus*
06***	Male	1.9	Sunflower oil changed to liquid paraffin	1.6 months; from week 1 of life; 5 mL orally thrice daily	No GOR‡	Cough, tachypnoea, hypoxia, digital clubbing, hyperinflation	4.3	Human metapneumovirus
					No aspiration§§§			
					Coughed and choked after getting oil‡‡‡			
07† ^,^ ††	Male	10.8	Olive oil	14 days; from age of six months; 2.5 mL orally twice daily	Failure to thrive	Cough, tachypnoea, hypoxia, digital clubbing	1.0	Parainfluenza 2 and 4*, *Streptococcus pneumoniae* *, *Moraxella catarrhalis* *, Adenovirus, human bocavirus, Enterovirus, RSV‐B
					No GOR‡			
					Coughed and choked after getting oil, fed oil while lying flat‡‡‡			
08‡‡ ^,^ §§	Male	4.3	Plant‐based “fish” oil¶¶¶	Exact details not known	None**	Cough, tachypnoea, hypoxia, crepitations	0.7	*Proteus mirabilis* *, human rhinovirus*, RSV‐B*, PCP oocysts*, human bocavirus, Coronavirus NL63
				Child under the care of grandmother	No aspiration§			
09† ^,^ ****	Male	3.7	Sunflower oil	Three months; from age two months; 2.5 mL orally thrice daily	GOR‡, silent aspiration§§§,renal disease††††, haemo‐dynamically insignificant 3 mm ASD	Cough, fever, tachypnoea, crepitations, bronchial breath sounds, one prior LRTI hospitalization	0.6	*Mycobacterium abscessus* *, *Klebsiella pneumoniae* *, *Acinetobacter baumannii* *, human rhinovirus, human bocavirus
10***	Male	2.1	Sunflower oil	Two months; from day 6 of life; 5 mL orally twice daily	GOR‡, silent aspiration§§§	Cough, tachypnoea, hypoxia, one prior LRTI hospitalization	0.1	None
11‡‡	Male	2.6	Olive oil	1.6 months; from age of four weeks; 2.5 mL orally once daily	No GOR‡,no aspiration§§§	Cough, fever, tachypnoea, hypoxia, reduced air entry on the right	1.4	*M. abscessus* *, *Pseudomonas aeruginosa* *, Candida species*
					Coughed and cried after getting oil‡‡‡			
12‡‡	Male	3.1	Plant‐based “cooking” oil†††	2.1 months; from age	None**	Cough, fever, tachypnoea, hyperinflation, crepitations, one prior LRTI hospitalization	0.4	RSV‐A*, *Klebsiella pneumoniae* *, *Bordetella pertussis*
					Coughed and cried after getting oil‡‡‡			

BAL, bronchoalveolar lavage; GOR, gastroesophageal reflux; LRTI, lower respiratory tract infection.

* Infection present in the BAL.

† Has sibling/s who also received oil and experienced respiratory‐related complications.

‡ Milk scan performed.

§ Swallowing assessed by speech therapist.

¶ Unable to reach patient on phone or in the community.

** Only clinical history at BAL diagnosis.

†† Siblings within the case series.

‡‡ Has no siblings.

§§ Child was left under the care of the grandmother.

¶¶ Posterior urethral valves (PUVs) ablated and bladder neck surgically excised.

*** Has sibling/s who also received oil but did not experience respiratory‐related complications.

††† Plant‐based oil, including blends of soya bean oil, sunflower oil, canola oil, or unspecified.

‡‡‡ Risk factor history from caregiver qualitative interview.

§§§ Contrast swallow performed.

¶¶¶ “Fish” oil is not oil from fish but rather a plant‐based oil used for frying fish among other foods.

**** Sibling death during infancy related to a similar respiratory illness.

†††† Dysplastic right kidney with cysts, enlarged left kidney with hydronephrosis, no PUVs, normal renal function.

‡‡‡‡ Retrospective diagnosis of ELP, child given oil for an additional six months.

### Radiological Characteristics

Plain chest radiographs were performed on all children. The initial radiographs taken at hospitalization in which a diagnostic BAL was performed showed diffuse ground‐glass opacification in all 12 cases. Predominantly right upper lobe expansile consolidation was noted in two of 12 participants. Nine of 12 children underwent high‐resolution computed tomography chest, and four distinct patterns were evident: (1) ground‐glass opacification with lower zone predominance (9/9); (2) smooth interlobular septal thickening (8/9)/crazy paving appearance (5/9); (3) expansile right upper lobe consolidation (2/9); and (4) fat attenuation within the areas of airspace consolidation (1/9). These patterns were present in various combinations.

### Pathological and Microbiological Characteristics

**Figure 1:**
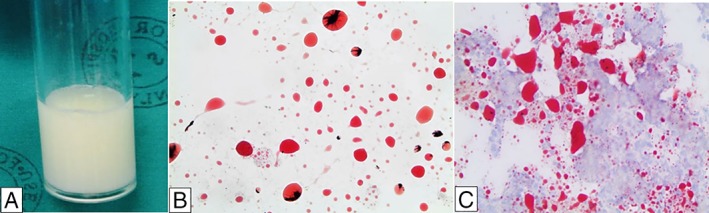
(A) Bronchoalveolar specimen macroscopic appearance: Milky–oily. (B) Bronchoalveolar specimen microscopy: Oil red O staining showing mainly large extracellular lipid droplets with isolated macrophages. (C) Frozen section lung biopsy microscopy: Oil red O staining showing large lipid droplets within the alveolar space.

Twelve BAL samples and one frozen section lung biopsy were assessed. On gross inspection, all BAL specimens were cloudy, 11 of 12 being predominantly milky in nature (Fig. 1). Histocytological assessment of most specimens revealed an abundance of fat‐laden macrophages and large extracellular droplets on Oil Red O staining. Eight children had BAL samples with neutrophil‐predominant type inflammation. Lung biopsy performed in one patient showed evidence of chronic lymphocytic interstitial inflammation in addition to large extracellular lipid droplets on the Oil Red O stain of the frozen section.

Only 3 of 12 children had negative BAL specimens on microbiology assessment; six of nine were polymicrobial. *Mycobacterium absessus complex* was cultured from BAL specimens in two children with normal immunological work up and expansile pneumonias involving the right upper lobe; *Mycobacterium abscessus boletti* was also isolated from blood culture after 108 h in one of these children.

### Treatment and Outcomes

Supportive treatment provided comprised: educating caregivers to stop administering oil to their children (7/12); oxygen supplementation (11/12), non‐invasive ventilation (8/12), and mechanical ventilation (1/12); antibiotics (12/12); and systemic corticosteroids (8/12). Five patients underwent partial therapeutic lung lavage. Four children needed only one procedure to obtain satisfactory clinical response, and one needed three sequential lavages over 11 weeks before achieving satisfactory clinical improvement. The median hospital stay duration was 23 (range 2–117) days. Clinical resolution was documented in 10 of 12, with a median time to clinical resolution from presentation of 1.1 (range 0.3–14) months. To date, radiological resolution on plain radiography from presentation is only documented in two, at 19.4 and 27.0 months, respectively. No mortalities were reported, and the children continue to be followed up in our service.

## Discussion

Exogenous lipoid pneumonia in children has been described in Asia, the Americas, and Europe 8, 9, 10. To the best of our knowledge, this is the first study from Africa describing histologically confirmed ELP. Our study reveals that this diagnosis may go unrecognized if a history of oil administration is not obtained in children presenting with recurrent/persistent pneumonia or ILD. Oil was administered to alleviate colic and constipation, consistent with findings from other regions 5, 8.

Furthermore, *M. abscessus* co‐infection has not previously been reported in children with ELP. Other Nontuberculous Mycobacteria (NTM) described in children with ELP include *Mycobacterium fortuitum*, *Mycobacterium chelonei,* and *Mycobacterium smegmatis* 8, 11. *Mycobacterium abscessus* complex has been reported in adult patients with ELP 12. It is postulated that oil increases the pathogenicity of mycobacteria possibly by hindering macrophage function and phagocytosis 3. Kudoh et al. demonstrated that there was increased virulence of NTM when inoculated in oil in comparison with aqueous solutions 13. Similarly, the lipid environment in the lung may be responsible for other secondary infections detected in BAL cultures among children with ELP 6, also seen in this series.

Diffuse ground‐glass opacification, predominantly in the posterior segments, and interlobular thickening were common patterns on chest CT in children with ELP consistent with the literature. Fatty attenuation within consolidation has also been described in children with ELP; however, we only observed this pattern in one child 9, 14. Interestingly, a unique pattern of a dense expansile right upper lobe consolidation was noted in two children with confirmed *M. abscessus* disease. This possibly reflects a severe form of lung oleoma/paraffinoma characteristically seen in adults 14, 15.

Discontinuing oil, treating infections, identifying underlying risk factors, and overall supportive care in line with consensus reviews 1, 2, 9, 14 was instituted for children in this study. In addition, we report success in utilizing corticosteroids and partial therapeutic lung lavage as additional treatment strategies for ELP consistent with other series 9. However, data to establish the effectiveness of corticosteroids and therapeutic lavage for ELP are limited 1, 2.

In conclusion, our case series highlights that ELP is an uncommon but serious condition that may occur in any global context with similar cultural practices. Obtaining a history of oil administration from caregivers, chest radiography and cytological analysis of BAL are sufficient to diagnose ELP. NTM co‐infection should be excluded in children with suspected ELP. Health education messages to highlight the risks associated with the cultural practice of oil administration are needed.

### Disclosure Statement

This study was approved by the University of Cape Town Human Research Ethics Committee (548/2017).
